# Life-Threatening Infectious Complications in Sickle Cell Disease: A Concise Narrative Review

**DOI:** 10.3389/fped.2020.00038

**Published:** 2020-02-20

**Authors:** Dominik Ochocinski, Mansi Dalal, L. Vandy Black, Silvana Carr, Judy Lew, Kevin Sullivan, Niranjan Kissoon

**Affiliations:** ^1^Department of Anesthesiology, University of Florida, Gainesville, FL, United States; ^2^Division of Pediatric Hematology/Oncology, University of Florida, Gainesville, FL, United States; ^3^Division of Pediatric Infectious Disease, University of Florida, Gainesville, FL, United States; ^4^Congenital Heart Center, University of Florida, Gainesville, FL, United States; ^5^Department of Pediatrics, University of British Columbia and BC Children's Hospital, Vancouver, BC, Canada

**Keywords:** sickle cell disease, infection, children, sepsis, prophylaxis, vaccination, critical care

## Abstract

Sickle cell disease (SCD) results in chronic hemolytic anemia, recurrent vascular occlusion, insidious vital organ deterioration, early mortality, and diminished quality of life. Life-threatening acute physiologic crises may occur on a background of progressive diminishing vital organ function. Sickle hemoglobin polymerizes in the deoxygenated state, resulting in erythrocyte membrane deformation, vascular occlusion, and hemolysis. Vascular occlusion and increased blood viscosity results in functional asplenia and immune deficiency in early childhood, resulting in life-long increased susceptibility to serious bacterial infections. Infection remains a main cause of overall mortality in patients with SCD in low- and middle-income countries due to increased exposure to pathogens, increased co-morbidities such as malnutrition, lower vaccination rates, and diminished access to definitive care, including antibiotics and blood. Thus, the greatest gains in preventing infection-associated mortality can be achieved by addressing these factors for SCD patients in austere environments. In contrast, in high-income countries, perinatal diagnosis of SCD, antimicrobial prophylaxis, vaccination, aggressive use of antibiotics for febrile episodes, and the availability of contemporary critical care resources have resulted in a significant reduction in deaths from infection; however, chronic organ injury is problematic. All clinicians, regardless of their discipline, who assume the care of SCD patients must understand the importance of infectious disease as a contributor to death and disability. In this concise narrative review, we summarize the data that describes the importance of infectious diseases as a contributor to death and disability in SCD and discuss pathophysiology, prevalent organisms, prevention, management of acute episodes of critical illness, and ongoing care.

## Introduction

Human hemoglobin is a tetramer comprising two alpha (α) and two non-α globin chains that envelop oxygen-carrying heme moieties. Normal hemoglobin is composed of hemoglobin A (95% of total hemoglobin), which contains two α-chains and two beta (β)-chains, hemoglobin A2, which is composed of two α-chains and two delta (δ)-chains (1–4%), and fetal hemoglobin, which consists of two α-chains and two gamma (γ)-chains (70–90% at birth, with a subsequent decline through the first 6 months of life).

The sickle cell disease (SCD) phenotype is the result of the substitution of valine for glutamine at the 6th amino acid position of the β-chain. Hemoglobin that incorporates this amino acid substitution is referred to as sickle hemoglobin (HbS). Patients who are heterozygous at this locus have sickle cell trait (HbAS) and are largely asymptomatic, whereas patients homozygous for the sickle β-chain mutation have sickle cell anemia (HbSS). Patients who inherit the sickle β-chain mutation along with other distinct β-chain mutations such as sickle β-thalassemia (HbSβ^0^ or HbSβ^+^ thalassemia) or hemoglobin C (HbSC disease) also exhibit the SCD phenotype. Patients with HbSS and HbSβ^0^ experience severe symptoms, whereas patients with HbSC and HbSβ^+^ are generally less affected.

**Figure 1:**
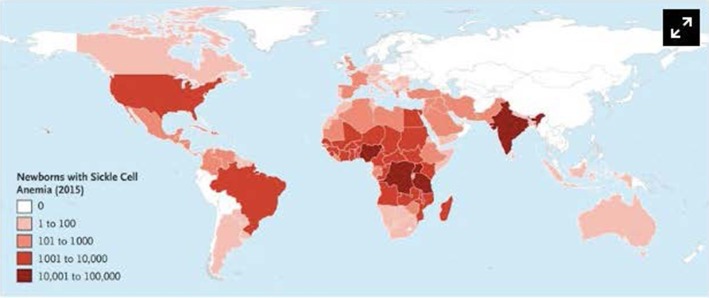
Number of newborns with Sickle Cell Anemia is Each Country in 2015. Data are based on estimates from Piel et al. Alaska is shown separately from the rest of the United States. Used with permission from Piel FB, Steinberg MH, Rees DC. Sickle cell disease. N Engl J Med (2017) 376:1561-1573.

SCD has a worldwide distribution. It is estimated that 300,000 infants are born annually with SCD, most in sub-Saharan Africa (1, 2). In the United States, 1 in 2,500 live births are afflicted with SCD (3), and it is estimated that 100,000 patients with SCD live in the United States (4). However, patients living in high-income countries (HICs) account for only 10% of the world's SCD population (5). The African continent, in particular, bears the burden of SCD, where the United Nations estimates that 12–15 million of the world's 25 million SCD patients live (6). Childhood mortality among SCD patients is highest between 6 months and 3 years of age (5–8), and it is estimated that 75% of all babies born with SCD are born in Africa where the mortality rate for children under 5 is estimated to exceed 50% (5, 9). The worldwide distribution of SCD is illustrated in Figure 1.

Immune function in pediatric SCD patients is impaired for a variety of reasons, including deficient splenic clearance of opsonized encapsulated bacteria (10, 11). This results in a propensity to infection by encapsulated bacteria such as *Streptococcus pneumoniae, Haemophilus influenzae, Salmonella typhi* and *non-typhi*, and *Meningococcal* species. In sub-Saharan Africa, one-half of patients with SCD die from infection before the age of 5, and children with SCD are >50 times more likely to suffer from invasive pneumococcal disease (12–16).

Early childhood SCD mortality has been dramatically reduced in HICs due to neonatal SCD screening, the provision of vaccination and prophylactic antibiotics, the aggressive early use of intravenous antibiotic therapy for febrile episodes, and the availability of contemporary pediatric critical care services. SCD patients in HICs commonly live into the fourth decade and beyond (17–19), during which time they accumulate chronic injury to their cardiovascular, central nervous, renal, pulmonary, and musculoskeletal systems.

SCD is a multisystem disease characterized by disordered hemoglobin structure, aberrant endothelial interactions, systemic inflammation, oxidant stress, and activation of the coagulation system. These derangements result in a tenuous physiology susceptible to infection-mediated acute crises, including splenic sequestration, acute chest syndrome, stroke, aplastic and vaso-occlusive crises, long-term disability, and death.

Herein, we discuss aspects of severe infections in children with SCD, including burden pathophysiology, prevention, therapy, and outcomes.

## Materials and Methods

The PubMed and Google Scholar databases were queried for English-language original research, literature reviews, systematic reviews, case reports, and meta-analyses relevant to the epidemiology, outcomes, prevention, treatment, and pathophysiology of infectious complications in SCD patients. Search terms included combinations of the following terms; sickle cell disease, sickle, chronic, organ dysfunction, hemoglobinopathy, pathophysiology, bacteria, bacterial, virus, viral, parasitic, malaria, sepsis, pneumococcal, invasive, *Haemophilus, Streptococcus, Salmonella*, HIV, tuberculosis, infection, complications, pneumonia, osteomyelitis, meningitis, bacteremia, vaccination, spleen, splenic, opsonization, prophylaxis, prevention, guidelines, recommendations, and immunization. Publications identified by the primary search were reviewed, and additional references were retrieved from the bibliographies of the articles identified by the primary search. The publications most appropriate to the pre-selected topics to be covered in this concise review were selected for inclusion.

## Predisposition to Infection

Patients with SCD are prone to infection for a variety of reasons that include splenic dysfunction, defects in opsonization of encapsulated organisms, impaired adaptive immunity, and immune deficiencies associated with malnutrition. The factors that contribute to immune deficiency in patients with SCD are summarized in Table 1. Indeed, malnutrition and the onset of splenic dysfunction early in life results in life-long deficiencies in innate, humoral, and cellular immune function.

**Table 1:** Summary of immune system dysfunction and mechanisms leading to increased susceptibility to infections in patients with sickle cell disease.

**System**	**Mechanism**
**INNATE IMMUNE DYSFUNCTION**	
Neutrophil dysfunction (20)	Impaired neutrophil chemotaxis, migration, and killing ability
Splenic dysfunction (21, 22)	Repeated sickling within the spleen leads to compromised splenic filtration of microorganisms
Reduced opsonization (23, 24)	Reduced opsonin production, leading to decreased ability to destroy encapsulated organisms
**ADAPTIVE IMMUNE DYSFUNCTION**	
Decreased humoral immunity (25)	Loss of splenic marginal zone leads to reduced number of Memory B cells and reduced antigen-specific immunoglobulin M secretion
Impaired virus-directed immunity (26)	Decreased Th1 response with reduced CD4+:CD8 suppressor T Cells
**MECHANICAL FACTORS**	
Increased susceptibility to osteomyelitis (27)	Bony infarction secondary to sluggish circulation leading to infarcts, which then act a nidus for bacterial proliferation
**NUTRITIONAL**	
Impaired virus-directed immunity (28, 29)	Zinc deficiency leads to lymphopenia and decreased Th1 response

### Splenic Dysfunction

The spleen is primarily responsible for filtering circulating pathogens and promoting innate and adaptive immune functions. Bacterial killing by macrophages is hampered because microbial opsonization with antibodies and/or complement, a prerequisite for the destruction of encapsulated bacteria, is impaired (21, 22, 30). Macrophages also present microbial antigens to T-lymphocytes, which in turn stimulate B-lymphocytes to produce high-affinity antibodies required for opsonization of encapsulated bacteria (31, 32).

Splenic dysfunction, including impaired filtration, deformation, and stagnation of red blood cells, and shunting, begins in infancy and is reflected by the presence of Howell-Jolly bodies in the peripheral blood (33). Chronic vaso-occlusion and ischemia damage the structure and function of the spleen, resulting in auto-splenectomy by 3–5 years of age, but functional asplenia and the resultant susceptibility to serious bacterial infection is present even earlier in childhood (34).

### Opsonization

Splenic opsonization is impaired due to deficient immunoglobulins and impaired production of the opsonins required for bacterial destruction. Efficient opsonization relies on the complement cascade, which is activated by classical and alternative pathways and kills invading microbes by inserting pores into their cell membranes (35, 36). In SCD, the insufficient availability of splenic immunoglobulin (Ig) M results in impaired opsonization and diminished classical pathway activation. Other opsonins, including tuftsin, which are produced in the spleen and needed for the activation of granulocytes, macrophages, and monocytes, are decreased in patients with SCD, suggesting a role for the spleen in their production (23, 24). Similarly, deficient levels of complement factor B, a protease that binds to C3b and amplifies the alternative complement pathway, is deficient, likely due to consumption from clearing sickled erythrocytes (37–39).

### Lymphocytes

Children with SCD are also vulnerable to atypical bacteria and viruses, suggesting other defects in immunity. B- and T-cell lymphocyte function are impaired in SCD, resulting in inadequate memory B-cell function and T-cell–independent production of natural anti-polysaccharide antibodies (25, 40, 41). The IgM antibody response to the influenza vaccine is also diminished in SCD patients (41). Circulating CD4+ and CD8+ T-lymphocytes are reduced in patients with SCD, and their differentiation into mature lymphocytes is adversely affected (26). This results in a diminished humoral immune response (referred to as Th2) or a cell-mediated immune response (referred to as a Th1 response) by CD4+ T-lymphocytes. Polarization of naive CD4+ cells away from an effective cell-mediated immunity effector stance may explain the development of severe influenza virus infections in children with SCD (42).

### Other Factors

Sluggish blood flow through the bone and bone marrow promotes bone ischemia, necrosis, and increased susceptibility to *Salmonella* osteomyelitis (27). Nutritional deficiencies impair the immune system in children with SCD. Deficiencies of macro- and micronutrients are present in children with SCD due to mechanisms that may include diminished caloric intake, elevated resting metabolic rate, increased red cell synthesis, elevated protein turnover, dysregulated inflammation, and increased myocardial energy demands (43). Micronutrient deficiencies have been implicated in increased susceptibility to infections and increased frequency of SCD-specific complications.

Low serum immunoglobulin levels are a commonly reported immune abnormality in malnourished children. Zinc deficiency develops as a result of poor dietary intake, high protein turnover, and increased losses from the kidneys due to inadequate reabsorption (44). Zinc deficiency has been linked to lymphopenia, reduced IL-2 production (required for adequate development of cell-mediated immunity), and is associated with deficient coordination of the innate and adaptive immune systems (28, 29). Zinc supplementation in SCD children has been demonstrated to improve somatic growth (45, 46), and supplementation of vitamins A, B, and magnesium has been demonstrated to decrease the frequency of infection, painful crisis, and emergency department visits (47, 48).

## Infectious Complications

Infectious pathogens of relevance to patients with SCD include bacteria, viruses, parasites, and mycobacteria. Table 2 presents a summary of the features of common infections seen in SCD patients. Empiric antibiotic treatment for bacterial infections are summarized in Table 3 and should be tempered by the local epidemiology and resistance patterns of bacterial pathogens.

**Table 2:** Most common pathogens in patients with sickle cell disease, including those living in austere environments.

**Infection/system**	**Micro-organism (s)**	**Complications/chronic organ dysfunction**	**Treatment**
Bacteremia/sepsis	*S. pneumoniae, S. aureus, GNR (Typhi and non-typhi Salmonella, E. coli, Klebsiella* sp., *H. influenzae type B), Bacteroides* sp. (49–51)	Septic shock with multi-organ failure (50, 52)	-*S. pneumoniae*, Gram-negative rods, some Bacteroides: third-generation cephalosporins (Ceftriaxone, Cefotaxime)^*^ - *S. aureus*: oxacillin, nafcillin, or cefazolin (MSSA)^*^; Vancomycin, clindamycin (MRSA)^*^
Meningitis/central nervous system infection	*S. pneumoniae, H. influenzae*, and *N. meningitides* (11, 52); *Pasteurella multocida* and *Capnocytophaga* sp. (in the presence of dog bite), viruses (Enteroviruses, herpes simplex viruses, mosquito-borne viruses); *Cryptococcus neoformans* and cerebral *Toxoplasma gondii* (especially in the presence of HIV)	Seizures, hemorrhagic stroke, acute ischemic stroke, venous sinus thrombosis, silent cerebral infarction, intra-cranial abscess, cognitive impairment (52–54)	- Third-generation cephalosporins (Ceftriaxone, Cefotaxime)^*^ - *Capnocyphaga* sp.: beta-lactam/beta-lactamase inhibitors and carbapenems (imipenem, meropenem) - *Pasteurella multocida*: penicillin (drug of choice) - *Cryptococcus neoformans*: a. Amphotericin B deoxycholate or liposomal amphotericin B + fluocytosine (induction phase), followed by fluconazole (consolidation therapy). - *Toxoplasma gondii*: pyrimethamine + sulfadiazine + folic acid. Allergy to sulfa: pyrimethamine + folic acid + clindamycin OR atovaquone - Herpes viruses: intravenous acyclovir
Upper and lower respiratory tract infection (sinusitis, epiglottitis, tracheitis, bronchitis, pneumonia)	Viruses (influenza viruses, respiratory syncytial virus, adenovirus, metapneumovirus, rhino-enterovirus, parvovirus B-19, parainfluenza viruses, cytomegalovirus, Epstein-Barr virus, and herpes simplex viruses, etc.) (55, 56); bacteria (*S. pneumoniae, H. influenzae, Chlamydophila pneumoniae, Mycoplasma pneumoniae, Legionella* sp., *S. aureus* (methicillin susceptible and resistant) (11, 57–59)	Acute chest syndrome, chronic lung disease/chronic restrictive lung disease, pulmonary hypertension	- Influenza: oseltamivir, inhaled zanamivir, baxtamivir - *S. pneumoniae, H. influenza type B*: third-generation cephalosporins (Ceftriaxone, Cefotaxime)^*^; *S. aureus*: oxacillin, nafcillin, or cefazolin (MSSA)^*^; Vancomcyin, clindamycin (MRSA)^*^ - Cytomegalovirus: intravenous gancyclovir/oral valganciclovir; Epstein-Barr virus: intravenous ganciclovir; Herpes viruses: intravenous or oral acyclovir, valacyclovir, or famciclovir - *Chlamydophila pneumoniae, Mycoplasma pneumoniae, Legionella* sp.: macrolides, quinolones
Musculoskeletal (skin and soft tissue infection, septic arthritis, fasciitis, myositis, osteomyelitis)	*Typhi* and *non-typhi Salmonella*, Gram-negative enteric bacteria, other Gram-negative (*Kingella kingae*, especially in the presence of negative cultures); *S. aureus* (methicillin susceptible and resistant), *S. pneumonia* (51, 52, 60–62)	Avascular necrosis, leg ulceration (skin), osteonecrosis (63, 64)	- Third-generation cephalosporins (Ceftriaxone, Cefotaxime)^*^ - *S. aureus*: oxacillin, nafcillin, or cefazolin (MSSA)^*^; vancomycin, clindamycin (MRSA)^*^ - *Kingella kingae*: ampicillin-sulbactam or a first-, second-, or third-generation cephalosporin
Gastrointestinal (cholelithiasis/choledocholithiasis, cholecystitis, cholangitis, intussusception), gastroenteritis	Enteric Gram-negative pathogens including *Typhi* and *non-typhi Salmonella*, Enterococci, anaerobic bacteria (65) and *Yersinia enterocolitica* infections (66)	Cholangiopathy (e.g., common biliary duct obstruction, cholestasis), hepatopathy (e.g., hepatic vaso-occlusive crisis, sequestration; hepatic fibrosis secondary to iron overload), mesenteric vaso-occlusion, and bowel infarcts	- Piperacillin-tazobactam or a carbapenem (imipenem, meropenem) - Surgical consult for decompression (stent/drains placement) or open/laparoscopic cholecystectomy - For Yersinia, a third-generation cephalosporin, piperacillin, or trimethoprim-sulfamethoxazole
Urogenital (urinary tract infection, pyelonephritis, renal abscess, urosepsis)	Gram-negative pathogens	Papillary necrosis, hematuria, renal failure, priapism (67)	- Third-generation cephalosporin (Ceftriaxone, Cefotaxime)^*^
Malaria	*Plasmodium falciparum, Plasmodium vivax, Plasmodium ovale, Plasmodium malariae*, and *P. knowlesi*	Vaso-occlusive crisis and secondary pain crisis, splenic sequestration; acute and chronic severe anemia requiring blood transfusion and causing folate-deficiency anemia; nephrotic syndrome, shock, hypoglycemia, acidosis, thrombocytopenia, and multi-organ failure	- Severe malaria requiring intensive care unit admission: intravenous quinidine until the parasite density <1% and able to tolerate oral therapy; alternative = intravenous artesunate^*2^ - Oral therapy: based on the infecting species, possible drug resistance, and severity of disease = Avoid exposure to mosquitoes and avoid areas with outbreaks of mosquito-borne infections.
Tuberculosis (*Mycobacterium tuberculosis*)	*Mycobacterium tuberculosis*	Vaso-occlusive crisis, acute chest syndrome; chronic pulmonary dysfunction, increased hemolysis, sub-optimal reticulocytosis, and anemia; extra-pulmonary tuberculosis (meningeal, lymph nodes, bones, joints, skin, middle ear, mastoid, gastrointestinal, renal)	- Presumed or known drug-susceptible pulmonary tuberculosis (except meningeal disease): a 6-month, 4-drug regimen consisting initially of rifampin, isoniazid, pyrazinamide, and ethambutol for the first 2 months and isoniazid and rifampin for the remaining 4 months - Drug-resistant tuberculosis: an expert in drug-resistant tuberculosis should be consulted for all drug-resistant cases
Human immunodeficiency infection	Human Immunodeficiency Virus (HIV-1 and HIV-2); HIV-2 is mainly prevalent in Western Africa	Increased risk for stroke, splenic dysfunction, avascular necrosis, and pulmonary arterial hypertension; increased risk of sepsis and bacterial infection, mainly pneumococcal infection	Because HIV treatment options and recommendations change with time and vary with occurrence of antiretroviral drug resistance and adverse event profile, consultation with a HIV expert is recommended^¥^.
Dengue fever, dengue hemorrhagic fever/dengue shock syndrome	Arbovirus (*Flaviviridae* family; genus *Flavivirus*)	Vaso-occlusive crisis, splenic sequestration, leg ulcers requiring amputation, myocarditis, heart block, shock, plasma leakage and secondary pulmonary and brain edema, ascites, anasarca, hemorrhage, multiorgan failure	- Supportive care: high intake of fluids, soft diet, acetaminophen [avoid salicylate-containing (aspirin) and non-steroidal anti-inflammatory products (ibuprofen)] and tepid sponging for relief of fever, adequate oxygenation, vasopressors, intravenous isotonic crystalloid (0.45% sodium chloride if <6 months of age) and colloids solution (avoid overload), blood products transfusion, diuretics for fluid overload; empirical therapy for bacterial infection pending cultures results. Steroids, anti-viral therapy (chloroquine, balapiravir, celgosivir).
Parasitic infections	Helminths: *Ascaris lumbricoides, Ancylostoma duodenale, Trichuris trichiura, Strongyloides stercoralis*, Schistosomiasis (*S. mansoni, S. haematobium, S. japonicum*), *Toxocara canis*, filariasis (*Onchocerca volvulus*)	- Vaso-occlusive crisis, chronic iron deficiency, chronic eosinophilia - Malnutrition, growth delay, cognitive deficit - Acute intestinal obstruction with intestinal perforation and peritonitis; appendicitis, common bile duct obstruction with secondary biliary colic, cholangitis, or pancreatitis (ascariasis) - Infiltrative eosinophilic pneumonitis syndrome (Ascariasis, Ancylostomiasis, schistosomiasis, filariasis, toxocariasis) with secondary hypoxia and acute chest syndrome	- *lumbricoides, A. duodenale*: albendazole, mebendazole, and pyrantel pamoate - *T. trichiura*: albendazole, mebendazole, and ivermectin - *S. stercoralis*: ivermectin (drug of choice), mebendazole - Schistosomiasis: praziquantel (drug of choice) - *T. canis*: albendazole, mebendazole - Filariasis: ivermectin; alternative = doxycycline (not recommended for children younger than 8 years)
		- Urinary schistosomiasis (*S. haematobium*) causing hematuria and predisposition to bacterial infection - Hepatosplenomegaly, bloody diarrhea, portal hypertension, ascites, esophageal varices, and hematemesis (chronic *S. mansoni* and *S. japonicum* infections) - Visual loss/blindness (filariasis, *T. canis*) - Strongyloides hyperinfection syndrome (in immunocompromised patients); larvae migration to distant organs causing fever, abdominal pain, diffuse pulmonary infiltrates, septicemia, and meningitis caused by enteric Gram-negative bacilli	
	Protozoa (other than malaria): *Entamoeba histolytica, Entamoeba coli* and *Giardia lamblia*	Chronic Giardia infection with secondary chronic intestinal malabsorption and failure to thrive - Toxic megacolon, fulminant colitis, ulcerations on colonic mucosa and secondary perforation; hepatic, pleural, lungs, and pericardium abscesses (*E. histolytica*)	- Hand hygiene after defecation, before preparing or eating food, after changing a diaper or caring for someone with diarrhea and after handling an animal or its waste - Sanitary disposal of fecal material - Treatment of drinking water (boiling, chemical disinfection with iodine or chlorine, use of filters) - Sexual transmission: use of condoms and avoidance of sexual practices that may permit fecal-oral transmission (*E. histolytica*). - Refrain from using recreational water venues (e.g., swimming pools, water parks) until asymptomatic and completed treatment

* *Antibiotic selection should take into consideration local epidemiology and antibiotic-resistant patterns. For developing countries, also refer to World Health Organization recommendations: https://apps.who.int/medicinedocs/en/d/Js5406e/*.

*2 *Available through the CDC malaria hotline investigational new drug (IND) protocol*.

¥ *http://aidsinfo.nih.gov*.

**Table 3:** Prophylaxis for the most common pathogens in patients with sickle cell disease, including those living in austere environments.

**Infection/system**	**Micro-organism(s)**	Prophylaxis^*^^+^
Bacteremia/sepsis	*S. pneumoniae, S. aureus*, GNR (*Typhi* and *non-typhi Salmonella, E. coli, Klebsiella* sp., *H. influenzae*), *Bacteroides* sp. (49–51)	- Diphtheria/tetanus/pertussis/*H. influenzae type B*/polio/13-valent pneumococcal vaccine at 2, 4, and 6 months (13-valent pneumococcal and *H. influenzae type B* also at 12–15 months); - *S. pneumoniae* 23-valent vaccine (at 2 years of age and 5 years later) at least 2 months after the 13-valent vaccine (68) - Penicillin V prophylaxis; erythromycin if penicillin allergy. Starting at age of 2 months until 5 years or for a history of pneumococcal sepsis or surgical splenectomy and continued lifelong (68) - *Salmonella typhi* vaccine for travel to resource-poor areas
Meningitis/central nervous system	*S. pneumoniae, H. influenzae*, and *N. meningitidis* (11, 52); *Pasteurella multocida* and *Capnocytophaga* sp. (in the presence of dog bite), viruses (Enteroviruses, herpes simplex viruses, mosquito-borne viruses); *Cryptococcus neoformans* and cerebral *Toxoplasma gondii* (especially in the presence of HIV)	- Diphtheria/tetanus/pertussis/*H. influenzae type B*/polio/13-valent pneumococcal vaccine/meningococcal vaccine^+^ at 2, 4, and 6 months (13-valent pneumococcal/*H. influenza type B* also at 12–15 months and meningococcal vaccine^+^ at 12 months). - *S. pneumoniae* 23-valent vaccine (at 2 years of age and 5 years later) at least 2 months after the 13-valent vaccine (68) - Meningococcal vaccine^+2^, including travel to resource-poor areas. - Meningococcal B vaccination^+3^, can start at 10 years of age. - Penicillin V prophylaxis; erythromycin if penicillin allergy. Starting at age of 2 months until 5 years or for a history of pneumococcal sepsis or surgical splenectomy and continued lifelong (68) - Avoid bites, scratches, and have mindful contact with dogs (*Capnocytophaga* sp., *Pasteurella multocida*).
Upper and lower respiratory tract infection (sinusitis, epiglottitis, tracheitis, bronchitis, pneumonia)	Viruses (influenza viruses, respiratory syncytial virus, adenovirus, metapneumovirus, rhino-enterovirus, parvovirus B-19, parainfluenza viruses, cytomegalovirus, Epstein-Barr virus, and herpes simplex viruses, etc.) (55, 56); bacteria (*S. pneumoniae, H. influenzae, Chlamydophila pneumoniae, Mycoplasma pneumoniae, Legionella* sp., *S. aureus* (methicillin susceptible and resistant) (11, 57–59)	- Annual influenza vaccine from 6 months. - Diphtheria/tetanus/pertussis/*H. influenzae type B*/polio/13-valent pneumococcal vaccine at 2, 4, and 6 months (13-valent pneumococcal and *H. influenzae type B* also at 12-15 months). - *S. pneumoniae* 23- valent vaccine (at 2 years of age and 5 years later) at least 2 months after the 13-valent vaccine (68) - Penicillin V prophylaxis; erythromycin if penicillin allergy. Starting at age of 2 months until 5 years or for a history of pneumococcal sepsis or surgical splenectomy and continued lifelong (68)
Musculoskeletal (skin and soft tissue infection, septic arthritis, fasciitis, myositis, osteomyelitis)	*Typhi* and *non-typhi Salmonella*, Gram-negative enteric bacteria, other Gram-negative (*Kingella kingae*, especially in the presence of negative cultures), *S. aureus* (methicillin susceptible and resistant), *S. pneumoniae* (51, 52, 60–62) *H. influenzae type B*	- Diphtheria/tetanus/pertussis/*H. influenza type B*/polio/13-valent pneumococcal vaccine at 2, 3, and 4 months (13-valent pneumococcal/H. influenza also at 12–15 months). - *S. pneumoniae* 23-valent vaccine (at 2 years of age and 5 years later) at least 2 months after the 13-valent vaccine (68) - Penicillin V prophylaxis; erythromycin if penicillin allergy. Starting at age of 2 months until 5 years or for a history of pneumococcal sepsis or surgical splenectomy and continued lifelong (68) - *Salmonella typhi* vaccine for travel to resource-poor areas.
Gastrointestinal (cholelithiasis/choledocholithiasis, cholecystitis, cholangitis, intussusception), gastroenteritis	Enteric Gram-negative pathogens, including *Typhi* and *non-typhi Salmonella*, Enterococci, and anaerobic bacteria (65)	- Prevention of vaso-occlusive crisis: avoid hypoxia, acidosis, hypothermia, infection, hypovolemia, etc.) - Decreased hemolysis/gallstone formation: hydroxyurea, ursodiol, low-fat diet - Prophylactic cholecystectomy? - *Salmonella typhi* vaccine for travel to endemic areas, known exposure to a carrier or laboratorian who works with *S. typhi*.
Urogenital (urinary tract infection, pyelonephritis, renal abscess, urosepsis)	Gram-negative pathogens	Prevention of vaso-occlusive crisis: avoid hypoxia, acidosis, hypothermia, infection, and hypovolemia.
Malaria	*Plasmodium falciparum, Plasmodium vivax, Plasmodium ovale, Plasmodium malariae*, and *P. knowlesi*	- Lifelong antimalarial chemoprophylaxis in patients living in malaria-endemic countries. - Travel to malaria-endemic areas: a. Avoid exposure to mosquitoes and avoid areas with outbreaks of mosquito-borne infections. b. Mosquito barriers: b.1. Wear Permethrin clothing that fully covers arms and legs and closed shoes, especially during early morning and late afternoon. b.2. Bed nets, screens, and nets tucked around strollers and other confined spaces where young children are placed. Insecticide-treated nets. b.3. Use Environmental Protection Agency-registered mosquito repellents. c. Avoid outdoor activities at dawn and dusk in malaria-endemic areas). d. Malaria chemoprophylaxis^□^
Tuberculosis (*Mycobacterium tuberculosis*)	*Mycobacterium tuberculosis*	- Annual tuberculin skin test or interferon-gamma release assay for HIV-infected persons and incarcerated adolescents. - Bacille Calmette-Guerin (BCG) immunization for those living in endemic areas, in U.S.: when risk of exposure is unavoidable and failure or unfeasibility of other control methods. - Latent tuberculosis infection identification and treatment.
Human immunodeficiency infection	Human Immunodeficiency Virus (HIV-1 and HIV-2); HIV-2 is mainly prevalent in Western Africa	- Safe sex practices. - Pre-exposure prophylaxis for men who have sex with men, heterosexual couples, and injection drug users^e^. - Post-exposure prophylaxis (sexual, other non-occupational exposure, occupational exposure)^e^. - Prevention of HIV transmission from infected pregnant mother-to-child^Ω^.
Dengue fever, dengue hemorrhagic fever/dengue shock syndrome	Arbovirus (Flaviviridae family; genus Flavivirus)	- Avoid exposure to mosquitoes and avoid areas with outbreaks of mosquito-borne infections. - Eliminate local mosquito breeding sites (elimination/drainage of receptacles for standing water), keep swimming pools, children's wading pools, and bird baths clean; clearing clogged rain gutters. - Mosquito barriers: a. Wear Permethrin clothing that fully covers arms and legs and closed shoes, especially during early morning and late afternoon. b. Bed nets, screens, and nets tucked around strollers and other confined spaces where young children are placed. Insecticide-treated nets. c. Use Environmental Protection Agency- registered mosquito repellents.- Avoid outdoor activities during daylight hours in Dengue-endemic areas. - No chemoprophylaxis available. - In May 2019, Dengvaxia was FDA approved for 9-45 year olds with laboratory-confirmed prior dengue virus infection. Other vaccine candidates are under clinical trials.
Parasitic infections	Helminths: *Ascaris lumbricoides, Ancylostoma duodenale, Trichuris trichiura, Strongyloides stercoralis*, Schistosomiasis (*S. mansoni, S. haematobium, S. japonicum*), *Toxocara canis, T. filariasis* (*Onchocerca volvulus*)	- Sanitary disposal of human feces (all parasitic infections). - Vegetables cultivated in areas where uncomposed human feces are used as fertilizer must be washed thoroughly and cooked before eating (*A. lumbricoides*). - Wear shoes to avoid contact with contaminated soil (*A. duodenale*). - Chemotherapy prophylaxis: albendazole, mebendazole) to pre-school and school-aged children in areas with >20% prevalence of infection (*A. lumbricoides, T. trichiura*). - Screening and treatment of high-risk groups (e.g., children, agricultural workers, and immigrants from endemic areas (*A. duodenale*). - *S. stercoralis* serology in all people with unexplained eosinophilia. - Elimination of the intermediate snail host in endemic areas; avoid contact with fresh water streams, rivers, ponds, or lakes in endemic areas (schistosomiasis). - Proper disposal of cats and dog feces, deworming of dogs and cats, covering sandboxes when not in use (*T. canis*). - Repellents and protective clothing (long sleeves and pants) to decrease exposure to black flies' bites during the day; community-wide mass ivermectin treatment: filariasis).
Parasitic infections	Protozoa (other than malaria): *Entamoeba histolytica, Entamoeba coli*, and *Giardia lambli*	Protozoa (other than malaria): *Entamoeba histolytica, Entamoeba coli*, and *Giardia lamblia*- Chronic Giardia infection with secondary chronic intestinal malabsorption and failure to thrive. - Toxic megacolon, fulminant colitis, ulcerations on colonic mucosa and secondary perforation; hepatic, pleural, lungs, and pericardium abscesses (*E. histolytica*). - *E. histolytica*: a. Asymptomatic cyst excreters (intraluminal infection): paromomycin or diiodohydroxyquinoline/iodoquinol. b. Invasive colitis or extraintestinal disease: metronidazole or tinidazole followed by diiodohydroxyquinoline/iodoquinol or paromomycin. c. Percutaneous or surgical aspiration of large liver abscesses. d. Piperacillin-tazobactam or Meropenem if peritonitis. - Giardiasis: metronidazole, nitazoxanide, or tinidazole. - Hand hygiene after defecation, before preparing or eating food, after changing a diaper or caring someone with diarrhea, and after handling an animal or its waste.
		- Sanitary disposal of fecal material. - Treatment of drinking water (boiling, chemical disinfection with iodine or chlorine, use of filters). - Sexual transmission: use of condoms and avoidance of sexual practices that may permit fecal-oral transmission (*E. histolytica*). - Refrain from using recreational water venues (e.g., swimming pools, water parks) until asymptomatic and completed treatment.

* *The standard vaccine series of childhood according to the American Academy of Pediatrics and the Advisory Committee on Immunization Practices should also be provided to all patients with sickle cell disease*.

+ *CDC-guided immunization schedule (for 2019) with notes for those with sickle cell disease: https://www.cdc.gov/vaccines/schedules/hcp/imz/child-adolescent.html*.

+2 *Menveo (groups A, C, Y, W-135) or MenHibtix (groups C, Y, and Haemophilus b Tetanus Toxoid conjugate). Menactra not given until child is 2 years old, with two doses given 8 weeks apart unless travel to country with endemic disease—can start at 9–23 months and given 12 (preferred) or 8 weeks apart*.

+3 *Can be given to adolescents 16–23 years of age at clinical discretion. Bexsero: two-dose series at least 1 month apart. Trumenba: three-dose series at 0, 1–2, and 6 months. Bexsero and Trumenba are not interchangeable, and the same product must be given for all doses used in a series*.

□ *www.cdc.gov/malaria/resources/pdf/treatmenttable.pdf*.

e *www.cdc.gov/hiv/risk/prep/index.html*.

Ω *https://aidsinfo.nih.gov/guidelines/html/3/perinatal/224/whats-new-in-the-guidelines*.

### Bacterial Pathogens in SCD and Associated Anatomical Sites

**Bacteremia and sepsis** are commonly detected in SCD patients. In some series, bacteremia accounted for 10–32% of febrile illnesses (49, 52, 69, 70). In Africa, bacteremia was found in 14–32% of children with SCD, a greater incidence than is observed in HICs (49, 69, 70). In contrast, in 165 Jamaican patients, only 6% of the episodes was caused by bacteremia, but 10% of the bacteremic patients died from their infections (71). Mortality from septicemia reaches 35–50% in infants and young children (50).

Prior to the use of augmented vaccination schedules and routine penicillin prophylaxis, *S. pneumoniae* caused the majority of cases of severe sepsis, followed by *Neisseria meningitidis, H. influenzae*, and *Escherichia coli* (11, 50, 72, 73). The epidemiology of bacterial infections and sepsis varies with geographic and socioeconomic circumstances, and includes consideration of *Staphylococcus aureus*, Salmonella species, and Bacteroides species, and other Gram-negative enteric bacilli (49, 51). Short- and long-term intravascular catheters are a common risk factor for bacteremia (74, 75).

Studies in several African countries showed Gram-negative bacteria can cause >50% of bacteremic episodes in children with SCD, with *Klebsiella pneumoniae* (25%), Salmonella *species* (12.5%), and other Gram negatives (25%) were detected more often than Gram-positive bacteria such as *S. aureus* (25%) and *S. pneumoniae* (6.3%) in one such study (49, 76, 77). The reasons for differing epidemiologic results in these studies are unclear but may be due to (a) higher carriage of other microorganisms, (b) liberal use of antibiotics before hospital admission, which influences culture results (mainly penicillin derivatives, often available without prescriptions), (c) difficulties of identifying the cause of infection by routine cultures or polymerase chain reaction in resource-limited settings and, (d) early extra-hospital deaths of children with fulminant infections with encapsulated bacteria (49, 76–80).

Taken together, these data suggest that bacteremia is common in SCD patients in all socioeconomic envirnonments. Bacteremia is associated with significant morbidity and mortality. It is essential to treat it with antibiotics that cover *Streptococcal, Haemophilus*, and *Salmonella* species, but it is also necessary to consider coverage for *Staphylococcal* species and Gram-negative enteric bacteria, depending on local data.

**Meningitis** is caused by *S. pneumoniae* (70–75% of cases), *H. influenzae*, and *N. meningitidis. Salmonella* species, *E. coli*, and other Gram-negative enteric bacteria also cause meningitis in children with SCD and must be considered in the differential diagnosis. Mortality has been reported as 10–20% for meningitis (11, 52). Meningitis in SCD generally presents and progresses similarly to meningitis in children <5 years of age without SCD (52). However, meningitis in children with SCD may predispose to stroke, a common complication of SCD (52–54).

**Pneumonia** is commonly due to *S. pneumoniae* in younger children, and *Chlamydia pneumoniae, Mycoplasma pneumoniae, Mycoplasma hominis*, and *S. aureus* (and to a lesser extent *Legionella* species) in older children and adults (11, 57–59). Pulmonary infection is one of the principal triggers for acute chest syndrome, and it is often difficult to distinguish patients with acute chest syndrome from those with pneumonia or those who have both. Of 670 acute chest syndrome episodes analyzed during a 4-year period, 45.7% had an unknown cause, 29.4% were attributed to infection, 16.1% to infarction, and 8.8% to fat embolism (57). In an analysis of 292 acute chest syndrome episodes in which a cause could be determined, infection was responsible for one-half of the cases, and of these, 25% were caused by a *Chlamydia* species, 22% were caused by *Mycoplasma* species, and 22% were caused by viruses (57).

**Osteomyelitis** occurs in between 0.5 and 16% in children and adults with SCD (60, 61, 63, 81). *Salmonella* species and *S. aureus* are the most common infectious etiologies of acute osteomyelitis (42–57%) in North America (52, 60). *Salmonella* species are the most common pathogen in West Africa and Saudi Arabia (52, 62). *Salmonella typhi* (the only encapsulated *Salmonella* species), *Salmonella non-typhi* species, Gram-negative enteric bacteria, and *S. aureus* can all cause osteomyelitis (64). It is often challenging to differentiate vaso-occlusive crisis with bone involvement from osteomyelitis on the basis of imaging or laboratory studies. Osteomyelitis commonly affects the diaphysis of the femur, tibia, or humerus. However, any bone can be affected, and it may be multifocal after hematogenous spread (60, 67, 81). The etiologic organism can be identified by blood culture, aspiration of bone lesion, or bone biopsy (67, 72). Septic arthritis may complicate osteomyelitis and osteonecrosis in children with SCD, and the causative bacteria are similar to those of osteomyelitis (65, 67). *Mycobacterial* infection and fastidious pathogens such as *Kingella kingae* (which can be pursued with molecular testing) may be the reason for sterile cultures.

**Urinary tract infection** (UTI) prevalence in SCD patients ranges from 6 to 26% and is more common in children with SCD than healthy children (82). Vaso-occlusion within the vasa rectae of the inner medulla causes ischemia, renal infarction, papillary necrosis, and scarring of the renal medulla, which promotes UTI (83). Many episodes of Gram-negative septicemia in SCD are secondary to UTI (82–84). In a recent U.S.-based study, UTI was a common reason for fever in SCD children (70). Thus, screening with urinalysis and urine culture during febrile illnesses is important in SCD children, particularly young children ≤2 years of age (70, 84, 85).

**Abdominal pain with fever** is common among patients with SCD and its prevalence increases with age and severity of hemolysis (83). Abdominal pain, nausea, vomiting, fever, and jaundice are common presenting symptoms of cholecystitis. In a recent cross-sectional, hospital-based study of SCD patients, 92.3% of patients with cholecystitis were older than 5 years (86). The pathogens of concern in cholecystitis include enteric Gram-negative pathogens, *Enterococci*, and anaerobic bacteria. Piperacillin-tazobactam and carbapenem antibiotics are considered first-line therapies. Surgical consultation is needed for decompression (surgical-, percutaneous-, or endoscopic retrograde cholangiopancreatography-placed stent/drains) or open or laparoscopic cholecystectomy. Abdominal pain with fever is also characteristic of infection with *S. typhi* (Typhoid fever), Dengue viruses (Dengue fever), and *Yersinia enterocolitica* (thought to be related to the unusual use of iron by this microorganism). *Y. enterocolitica* has been associated with intussusception in a patient with sickle cell anemia (66).

**The oral cavity** is a source of infectious and non-infectious complications. Orofacial pain in SCD patients may be due to vaso-occlusion in the facial bones and dental pulp, or osteomyelitis of the facial bones. Osteomyelitis is more likely to occur in the mandible due to the relatively poor blood supply in this area. Pathogens responsible for facial osteomyelitis come from the gastrointestinal tract (cholecystitis or gastroenteritis) by hematogenous spread (87). *Salmonella* is a common etiology for facial osteomyelitis, but mixed flora and *S. aureus* also account for a substantial percentage of cases (87, 88). It is often difficult to differentiate early bone infarction from osteomyelitis. Blood culture, magnetic resonance imaging, and bone aspiration/biopsy may be diagnostic. Colonization of the oropharynx with *non-albicans Candida* species and unusual fungi has been observed in patients with SCD, suggesting that penicillin may affect the balance of oral flora (89).

### Viral Pathogens in SCD

Respiratory viruses (e.g., respiratory syncytial virus, influenza viruses, rhinovirus, human metapneumovirus, parainfluenza viruses) can trigger significant complications in patients with SCD, including acute chest syndrome, bacterial superinfection, aplastic crisis, splenic sequestration, and painful vaso-occlusive crisis (55, 57, 90). Viral respiratory pathogens promote the development of acute chest syndrome by inducing lung inflammation, injury to the microvasculature of the lung, airway hyper-reactivity, mismatch of ventilation and perfusion, and in some instances, secondary bacterial infection (commonly with *S. aureus* or *S. pneumoniae*) (91–93).

Other viruses such as Parvovirus B19, hepatitis B, hepatitis C, Epstein-Barr Virus, influenza, dengue, and human immunodeficiency virus (HIV) cause significant morbidity for SCD patients worldwide (11, 50, 56). Parvovirus B19 causes transient aplastic crisis in 65–80% of infections in SCD patients. It specifically infects erythroid progenitor cells, resulting in a temporary cessation of erythropoiesis and leading to severe anemia (94). Parvovirus B19 has also been associated with the development of acute chest syndrome, splenic and hepatic sequestration, bone marrow necrosis, pain crisis, and stroke (94). Epstein-Barr Virus infection can cause splenic rupture, thrombocytopenia, agranulocytosis, hemolytic anemia, and hemophagocytic lymphohistiocytosis in SCD. SCD individuals are at high risk for complications from influenza infections; they are hospitalized for influenza at a rate 56 times that of children without SCD (56). The liver may be adversely affected by hepatitis B and C, and HIV infections (72). In resource-challenged areas of the world, the blood supply is a major source of these infections, but unsafe injections given by untrained/informal providers and surgical practices such as circumcision and female genital mutilation are additional sources of hepatitis C transmission (95). The worldwide prevalence of hepatitis C and B infections among SCD patients ranges from 2 to 30% (96) and 1.5–18.9%, respectively (96, 97).

The prevalence of HIV seropositivity in SCD patients varies between 0 and 11.5% (98). Few data are available regarding the impact of coexistent HIV infection and SCD but both diseases increase the risk for stroke, splenic dysfunction, avascular necrosis, and pulmonary arterial hypertension (98). SCD patients with HIV may be more susceptible to infection with encapsulated bacteria as well as opportunistic pathogens. In a U.S. hospital-based study, SCD with HIV infection conferred a greater risk for hospitalization for bacterial infection and sepsis but less risk for vaso-occlusive crisis. Inpatient case fatality data for children with SCD and HIV were not different from that of children with SCD alone but fatalities were lower than those of children with HIV infection only (99). SCD may confer protection against HIV infection because of upregulation of inflammation, iron metabolism, and auto-splenectomy, which are not favorable for HIV replication (98).

A final concern emerging among viral pathogens is the significant overlap of the mosquito-borne dengue viruses (DENV 1–4) in geographic areas of the world where SCD is endemic. These include the Caribbean, Central and South America, areas of Africa and the Middle East, Asia, and Oceania (100, 101). Dengue hemorrhagic fever is a viral infection that is characterized by headache, fever, abdominal pain, bleeding, myalgias, and loss of capillary integrity with extensive third-space fluid losses, resulting in hypovolemia and death. Sickle cell patients, because of their intolerance of hypovolemia and proclivity to endothelial cell activation, are at increased risk to die from this viral infection (101, 102).

### Parasites

Malaria is caused by a protozoan parasite from the Plasmodium family that is transmitted by the bite of the Anopheles mosquito, a contaminated needle, or a blood transfusion. The sickle cell trait is believed to confer a protective effect against severe, life-threatening malaria because sickled erythrocytes are readily cleared by splenic macrophages (11, 72, 103). The effect of malaria on morbidity and mortality in homozygous SCD patients however is severe, with mortality in the SCD population significantly increased when compared to malaria in persons without SCD (104, 105). Studies in two African nations demonstrated that the incidence of malaria was not increased among patients with SCD, but that the risk of death was much higher in malaria patients with SCD when compared to those without SCD (106–108).

Parasitic infections are a common problem in developing countries, and can increase morbidity in patients with SCD. In a Nigerian study of 100 SCD patients, 27% were found to be infected with intestinal parasites that included four helminths (*Ascaris lumbricoides, Ancylostoma duodenale, Trichuris trichiura*, and *Strongyloides stercoralis*) and three protozoa (*Entamoeba histolytica, Entamoeba coli*, and *Giardia lamblia*) (109). Intestinal parasites increase the severity of anemia and the need for transfusion (109). Finally, urinary schistosomiasis is an endemic disease in rural and urban communities in Africa. Schistosomiasis infection in patients with SCD is associated with increased reticulocyte count and lower hematocrit due to urinary blood loss. In addition, urinary schistosomiasis can promote secondary bacterial UTI (110).

### Mycobacteria

*M. tuberculosis* and SCD are prevalent co-morbidities in austere environments, and features of tuberculosis in children with SCD are comparable to those in the general population with favorable outcomes with standard treatment (111).

## Infection Prophylaxis

Perhaps the greatest reductions in infectious morbidity and mortality have occurred as a result of advances related to the administration of antibiotic prophylaxis, vaccination, and the prompt administration of parenteral antibiotics during febrile illness. Indeed, the rationale for the perinatal screening of infants for SCD is to facilitate participation in these preventive measures. Table 3 summarizes the recommended antibiotic prophylaxis and vaccination schedules for the prevention of infections in SCD patients.

Before the advent of penicillin prophylaxis, the incidence of invasive pneumococcal disease was 6 episodes/100 patient years, with a peak in the first 3 years of life. Pneumococcal polysaccharide vaccine markedly reduces the risk of invasive pneumococcal disease in children receiving daily prophylactic penicillin (112). However, whether prophylaxis should be continued throughout adulthood is uncertain. Most pediatric hematologists recommend stopping prophylaxis at 5 years of age (112–114). Similarly, the role of penicillin prophylaxis in patients with HbSC, HbS-β^+^ thalassemia, and other compound heterozygotes is controversial. Current evidence-based guidelines from the National Heart, Lung, and Blood Institute (115) recommend oral penicillin prophylaxis [(125 mg for age <3 years and 250 mg for age 3 years and older) twice daily until 5 years of age in all children with homozygous SCD (Hb SS)]. These guidelines endorse the discontinuation of penicillin at 5 years of age if there is no history of invasive pneumococcal disease or surgical splenectomy and pneumococcal vaccination is adequate (115).

The heptavalent pneumococcal conjugate vaccine (PCV7) introduced in 2000 led to a further 70% decrease in the incidence of invasive pneumococcal disease (116), with more recent studies suggesting that the PCV13 vaccine introduced in 2010 has further reduced the incidence of serious pneumococcal disease (117, 118). Current standard practice should include the initiation of daily prophylactic penicillin by 2 months of age and the completion of the pneumococcal vaccine series (both PCV13 and pneumococcal 23-valent vaccine) by 5 years of age before the discontinuation of prophylactic penicillin. Some centers recommend pneumococcal polysaccharide vaccine boosters every 5 years, although this practice has not been endorsed by any set of evidence-based guidelines (68). In addition to standard immunizations recommended by the Advisory Committee on Immunization Practices, children with SCD should also be immunized against meningococcal disease and receive the annual influenza vaccine.

Because of the risk of invasive pneumococcal disease, any fever (typically defined as 38.5°C or higher) is treated as a medical emergency in children with SCD. National Heart, Lung, and Blood Institute Guidelines (115) recommend the urgent evaluation of all febrile episodes, including physical examination, complete blood count, and blood culture. Hospitalization for observation is sometimes necessary but most patients with SCD evaluated for fever without a source who lack certain high-risk features (white blood cell count >30,000/mm^3^ or <5,000/mm^3^, fever >40°C, “ill-appearing”) can be managed safely as an outpatient after intravenous administration of an empiric, anti-pneumococcal antibiotic that also provides Gram-negative enteric coverage (e.g., ceftriaxone) as long as other SCD-related complications (such as acute chest syndrome) have been excluded. The average time to positive blood cultures for children with SCD and bacteremia is <24 h (119), thus a single dose of ceftriaxone is probably sufficient in most cases of outpatient management.

In low- to middle-income countries, provision of prophylaxis for endemic infections, including malaria and dengue, should be considered. SCD patients traveling to or living within areas endemic for malaria and dengue fever would benefit from meticulous use of maximal mosquito protection techniques and malaria chemoprophylaxis (101, 105). Additionally, malaria prophylaxis should be considered for patients with SCD visiting endemic regions (105, 120). Long-term malaria chemoprophylaxis has been shown to lower the incidence of crisis and to reduce mortality, but few studies have evaluated its benefit, particularly in SCD (106). A recent Cochrane review reported that malaria prophylaxis reduces the frequency of sickle cell crisis, hospital admission, blood transfusion, and anemia severity but suggested further studies to compare antimalarial prophylaxis medications and to better characterize potential adverse outcomes of long-term prophylaxis (120). It is not known how antimalarial drug resistance affects its efficacy (121). New consensus guidelines for chemoprophylaxis of SCD remains a priority.

Two *Salmonella typhi* vaccines (oral live-attenuated Ty21a and inactivated Vi capsid polysaccharide for persons >6 and >2 years of age, respectively) are available in the United States typically for travelers to endemic typhoid fever areas. Their efficacy in persons with SCD is unknown (122, 123). Both vaccines are allowed for indication, since for vaccine purposes, persons with SCD are considered to have a medical condition with limited immune deficit (asplenia) (Centers for Disease Control and Prevention: https://wwwnc.cdc.gov/travel/yellowbook/2020/travelers-with-additional-considerations/immunocompromised-travelers). However, if age appropriate, the oral vaccine is recommended over the injectable vaccine, which has more known side effects. A recent phase 3 trial of a typhoid conjugate vaccine in Nepal demonstrated excellent immunogenicity, significantly reduced *Salmonella typhi* bacteremia, and had a similar frequency of adverse events as the group A capsular antigen Meningococcal vaccine that was used as a control vaccine (124).

A summary of considerations for persons with SCD traveling to or living in austere environments is also provided in Tables 3, 4. The United States Centers for Disease Control (https://wwwnc.cdc.gov/travel/yellowbook/2020/travelers-with-additional-considerations/immunocompromised-travelers) provides a website and book for travel medicine specialists that offers guidance for vaccination and medical prophylaxis for prevalent and endemic infections based on the location of rural or urban destination, recent outbreaks, length of stay, age and immune competency of the traveler, and other variables.

**Table 4:** Recommendation for patients with sickle cell disease traveling to austere countries^*^.

**Issue**	**Recommendations**
Vaccination^†^ (see also Table 3)	Ensure all age-appropriate vaccinations in Table 3: • *Streptococcus pneumoniae* (within 5 years) • *Neisseria meningitidis* (within previous 3 years) • *Haemophilus influenzae* B (once) • Influenza (annually) Additional vaccinations: • Yellow fever (if traveling to endemic African/American countries) • *Salmonella typhi* vaccination (if traveling to endemic areas)
Mosquito-borne illnesses (malaria/Dengue) (see also Table 3)	• Ensure basic preventive methods (mosquito nets, insect repellants, avoiding marshy/wet areas with mosquito habitats) • Chemoprophylaxis (all major prophylactic regimens are acceptable) • Prompt diagnosis and treatment of suspected malaria
Gastroenteritis/enteritis (see also Table 3)	• Avoid contaminated food and water • Employing adequate hand washing prior to eating or preparing food, after using bathroom • Carry oral electrolyte replacement solutions to ensure proper hydration and to avoid hypovolemia/vaso-occlusive crisis in the setting of gastroenteritis
Invasive bacterial infection(see also Table 3)	• Prophylaxis for duration of travel (amoxicillin 250–500 mg PO BID) • Seek medical advice promptly for management of febrile illness • Standby treatment of febrile illness (amoxicillin 3 g PO vs. fluoroquinolone)

* *See Willen et al. (125)*.

*Watson (126)*.

† *The standard vaccine series of childhood according to the American Academy of Pediatrics and the Advisory Committee on Immunization Practices should be provided to all sickle cell patients*.

Vaccination and penicillin prophylaxis are effective. In developed nations where children with SCD are identified by newborn screening programs and receive recommended prophylactic treatment with penicillin and an augmented immunization schedule, rates of bacteremia have been shown to be <1% (127). In resource-challenged socioeconomic environments, the priorities remain: (a) clarification of the spectrum of bacterial pathogens relevant to SCD patients in specific geographic regions, (b) implementation of comprehensive perinatal SCD screening, and (c) implementation of vaccination and antibiotic prophylaxis programs appropriate for the bacterial epidemiology of the region.

## Treatment of Life-Threatening Infections

Bacteremia presents on a continuum of severity that ranges from indolent to fulminant. The incidence of bacteremia varies from 6 to 25% and progresses to life-threatening disease in 10–25% of children in reports from both HICs and low- to middle-income countries.

These data suggest that bacteremia is not the most common cause of fever in persons with SCD and remains life threatening in a significant percentage of patients despite aggressive treatment. It is reasonable to assume that deficiencies in vaccination, antibiotic prophylaxis, rapid hospital transport, intravenous antibiotics, blood supply, microbiologic diagnostic techniques, and critical care capacity in austere environments serve to increase morbidity and mortality from bacteremia. Indeed, mortality rates of 35–50% from septicemia have been described (50).

### Infection and the Pathophysiology of SCD

**Figure 2:**
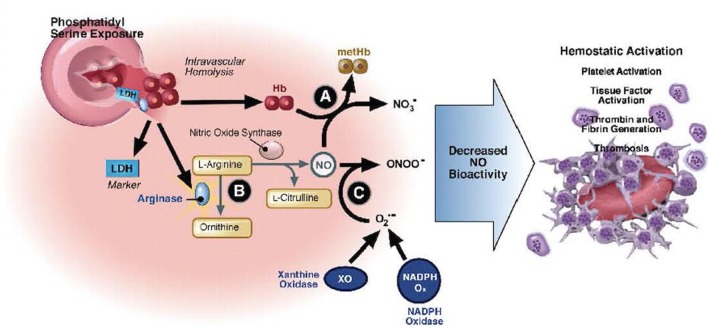
Hemolysis-associated hemostatic activation. Intravascular hemolysis releases hemoglobin into plasma which quenches nitric oxide (NO) and generates reactive oxygen species (directly via fenton chemistry or via induction of xanthine oxidase and NADP oxidase). In addition, arginase I is released from the red blood cell during hemolysis and metabolizes arginine, the substrate for NO synthesis, further impairing NO homeostasis. The depletion of NO is associated with pathological platelet activation and tissue factor expression. Hemolysis and splenectomy are also associated with phosphatidylserine exposure on red cells which can activate tissue factor and form a platform for coagulation. Used with permission from Gladwin MT, Kato GJ. Hemolysis-associated hypercoagulability in sickle cell disease: the plot (and blood) thickens! Haematologica (2008) 93:1-3.

Deoxygenation of HbS alters the structure of the β-chain of hemoglobin, resulting in reduced solubility, hemoglobin polymerization, and diminished membrane flexibility (128–130). Distortion of globin chains and exposure of intracellular heme iron intensifies intracellular oxidant stress, cell membrane damage, and erythrocyte dehydration (129, 130). Vascular occlusion, tissue ischemia, and a cascade of systemic inflammation activate endothelial cells to interact with erythrocytes, activated leukocytes, the coagulation cascade, and activated platelets (131–143). Potent vasoconstrictors are liberated from the endothelium in response to injury (131–145). These events result in endothelial dysfunction, inflammation, and tissue ischemia, which correlates with SCD symptom severity (146, 147). The clinical presentations of SCD-specific complications and serious infections can overlap. Care of the critically ill SCD patient must address infection while supporting the underlying pathophysiology of the disease to prevent or mitigate SCD-related complications. These interactions are summarized in Figure 2.

**Figure 3:**
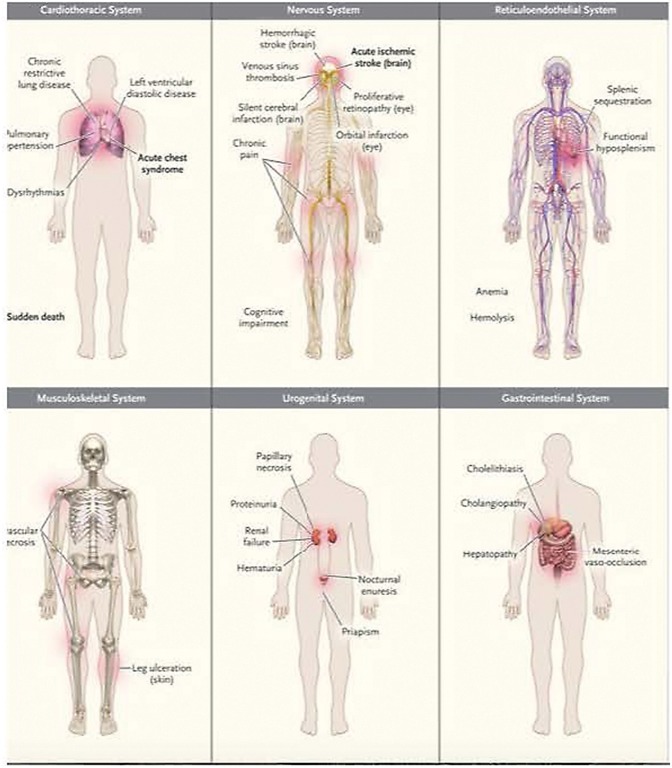
Common clinical complications of sickle cell disease. Data are from Rees et al. and Serjeant. Acute complications are shown in boldface type. Used with permission from Piel FB, Steinberg MH, Rees DC. Sickle cell disease. N Engl J Med (2017) 376:1561-1573.

Chronic endothelial inflammation and dysfunction cause progressive vital organ system deterioration over time. Relevant organ system considerations for critical care management are summarized in Figure 3.

SCD produces changes in the **cardiovascular system** of children, including diastolic dysfunction and left and right ventricular dilation, usually with preserved systolic function (148–150). Left ventricular systolic function may be impaired in adulthood in patients with renal disease and longstanding hypertension (151). Pulmonary artery hypertension develops for reasons that are multi-factorial and may reflect the intrinsic elevation of pulmonary vascular resistance or left ventricular dysfunction with normal pulmonary vascular resistance. The development of chronic pulmonary hypertension is an ominous finding associated with the increased occurrence of multi-organ system failure and premature mortality (152–155).

The **pulmonary system** is adversely affected by SCD. Younger patients may have mild restrictive lung disease, whereas adults with SCD can develop a severe form of restrictive lung disease referred to as sickle cell chronic lung disease. Asthma carries great clinical significance in SCD as its presence is associated with more frequent episodes of acute chest syndrome early in life and increased mortality and reduced lifespan in adulthood (156, 157).

The **renal system** suffers repeated ischemic insults in the hypertonic medulla that result in impaired urinary-concentrating ability (158). Dialysis and renal transplantation are sometimes necessary in adulthood. The intensivist managing critically ill SCD patients must be mindful to maintain hydration, intake and output, acid-base balance, and the renal elimination of medications. Continuous renal replacement therapy is often helpful in this regard.

The **central nervous system** is the vital organ system most impaired in childhood. The risk of stroke in SCD is highest in the first decade of life, with an incidence of 1% per year between the ages of 2 and 5 (159). Most strokes in children are ischemic, and hemorrhagic stroke accounts for up to one-third of strokes in adults (160). Increased transcranial Doppler flow velocities in the middle cerebral arteries are predictive of future strokes, and patients with increased transcranial Doppler, prior overt stroke, or silent cerebral infarcts are treated with chronic transfusion therapy to prevent future strokes. Risk factors for the recurrence of stroke include the presence of silent cerebral infarction, non-compliance with chronic transfusion therapy after a first stroke, presence of Moyamoya vasculopathy, acute decrease in hemoglobin concentration, history of transient ischemic attack, recent or recurrent episodes of acute chest syndrome, severity of anemia, and systolic hypertension (159, 161).

### Differential Diagnosis of Fever and Infectious Syndromes in SCD

**Fever without a source** is a common problem in SCD patients and should be regarded as a medical emergency requiring rapid evaluation and initiation of intravenous antibiotics. The presentation of sepsis is similar to children without SCD. The presentation of bacteremia may be subtle, however, and can include sudden fever, few prodromal features with a relatively well appearance, and then rapid deterioration sometimes associated with adrenal hemorrhage, progressive shock, and death (52).

Leukocytes and platelet counts may mimic findings in infections and are similar to children without SCD. An increased hematocrit can signify dehydration due to poor fluid intake or increased fluid losses, whereas decreased hematocrit may result from infection-induced hemolysis, acute chest syndrome, splenic sequestration crisis, malaria, or viral suppression of the bone marrow. Splenomegaly or hepatomegaly may indicate splenic or hepatic sequestration, malaria, or a variety of viral infections.

Diagnostic testing should be guided by the clinical history and physical examination. In the absence of localizing signs or symptoms of infection, blood cultures, urinalysis, and urine culture should be considered (especially in infants and sexually active females). Viral pathogens (i.e., enterovirus, influenza, and others) may also cause severe systemic disease, and molecular diagnostic panels for viral pathogens may also be appropriate. Further infectious testing should be guided by signs and symptoms. Blood cultures, as well as all other clinically indicated culture materials, should be obtained quickly and antibiotic administration should be expedited. Thorough physical examination should seek a focus of infection (indwelling intravascular catheter tunnel sites, facial and long bones, dental sources, and skin/soft tissue infections) that may require source control.

All febrile children should be evaluated for evidence of early focal infection of the respiratory system, central nervous system, abdomen, and musculoskeletal systems as well as SCD-specific complications, including acute chest syndrome, stroke, vaso-occlusive crisis, aplastic crisis, splenic sequestration, and hepatic sequestration.

**Fever with respiratory symptoms** such as cough, tachypnea, hypoxemia, fever, and chest pain may be due to pneumonia, acute chest syndrome, or both. If intrathoracic infection is suspected, sputum should be sent for bacterial culture, and polymerase chain reaction should be performed for viral detection. Adequate sputum specimens are difficult to obtain from young children who are not intubated but they can be induced from older children or may be obtained by bronchoscopy. Isolation of a predominant bacterial pathogen in the presence of a granulocytic response on Gram stain suggests the bacterial etiology, and the absence of such findings may suggest a mycobacterial infection or non-bacterial cause such as acute chest syndrome. Pleural fluid, if significant, should be sent for cell count, chemistries, pH measurement, bacterial Gram stain, and culture. In the setting of acute chest syndrome and/or pulmonary infection, *Staphylococci, Streptococci*, atypical bacteria, and respiratory viruses (respiratory syncytial virus, human metapneumovirus, rhinovirus, parvovirus, parainfluenza viruses, influenza viruses, cytomegalovirus, Epstein-Barr Virus, and Herpes simplex viruses) comprise a major source of infectious etiologies. Sputum acid-fast staining and Mycobacterial cultures should be considered in the appropriate clinical settings.

**Fever with central nervous system symptoms** occurs less commonly in SCD and is a medical emergency. Children with a central nervous system infection (meningitis or meningoencephalitis) may present with nuchal rigidity, photophobia, fever, and/or signs of elevated intracranial pressure. Alternatively, they may present with less definitive findings such as seizure, altered sensorium, or headache. Central nervous system differential diagnoses include ischemic or hemorrhagic stroke, seizure, and central nervous system infection. If central nervous system disease is suspected, lumbar puncture should be performed after neuroimaging has excluded the presence of markedly increased intracranial pressure, an alternate diagnosis (hemorrhagic or ischemic stroke), and coagulation status has been confirmed as normal. Administration of antimicrobial therapy should not be delayed for neuroimaging or if lumbar puncture has to be delayed.

Cerebrospinal fluid should be analyzed for cell count, differential, chemistries, and Gram stain, and bacterial culture and molecular diagnostic testing should be performed for bacterial and viral pathogens. Bacterial pathogens likely to be present include *S. pneumoniae, H. influenzae*, or *N. meningitidis*. Gram-negative pathogens occur occasionally and especially in neonates. In the presence of a dog bite, *Capnocytophaga* and *Pasteurella multocida* species should be considered. Bacterial pathogens and viral pathogens may be tested by polymerase chain reaction on cerebrospinal fluid. In the presence of appropriate risk factors, geographic location, and travel history, further testing for tuberculous meningitis, cerebral malaria, HIV and HIV-associated central nervous system infections (HIV, *Cryptococcus neoformans, Toxoplasma gondii*), fungal pathogens, roundworm infections, and amoeba should be considered. Empiric treatment with vancomycin, ceftriaxone, and acyclovir, if clinically indicated, are recommended. Empiric initial antibiotic therapy with vancomycin is recommended when *Staphylococci* or resistant *Streptococci* are in the differential diagnosis. Anti-pseudomonal antibiotics can be considered if nosocomial or neurosurgical infection is considered. Antiviral, antimalarial, and other therapies may be added based on risk factors, lab testing, and infectious disease consultant recommendations.

Regardless of the site of infection, if appropriate risk factors are present (recent hospitalization, inpatient status in a chronic care facility, prolonged broad-spectrum antibiotic or steroid use, or other causes of immune suppression), it may be prudent to consider empiric fungal coverage. Finally, in endemic areas, testing, and treatment for dengue viruses, malaria, tuberculosis, and HIV may be considered. Input from infectious disease and hematology specialists is essential.

### Approach to Antimicrobial Coverage

Appropriate initial empiric antibiotic therapy for the most likely pathogens is presented in Table 2. Initial antibiotic selection for patients presumed to be bacteremic should be broad and active against encapsulated organisms as well as other common pathogens. Antibiotic selection is further influenced by local epidemiology and antibiotic resistance patterns (162, 163). The empiric use of vancomycin and a third-generation cephalosporin provides coverage against: resistant pneumococci, meningeal penetration, and Staphylococci and enteric Gram-negative pathogens. Antibiotic coverage should be tapered based on culture results to minimize development of bacterial resistance and opportunistic fungal infection. The addition of anti-pseudomonal antibiotics should be considered if hospital-acquired pneumonia is present, and oseltamivir should be administered if influenza is considered likely.

### Critical Care Considerations in SCD

An exhaustive discussion of critical care support of sepsis with organ dysfunction is beyond the scope of this review. Critical care support of patients with severe sepsis and organ dysfunction is similar to that provided to patients without SCD with additional considerations specific to persons with SCD.

In this section, we will review the considerations relevant specifically to the management of SCD patients with severe sepsis. We present issues related to (a) hemodynamic assessment and support, (b) respiratory considerations, (c) the role of transfusion therapy in critically ill persons with SCD, and (d) precautions related to granulocyte colony-stimulating factor and coagulation factor replacement.

**Initial hemodynamic evaluation and management** should consider the patient's pre-morbid SCD-related circulatory system changes. The hemodynamic profile of the SCD patient with infection and shock may vary with respect to contractility and atrial filling pressures. Systemic resistance may be high, low, or normal, and pulmonary vascular resistance may be elevated. Shock states may be caused by systolic and/or diastolic dysfunction of either or both ventricles. The hemodynamic profile can change as infection progresses, necessitating re-evaluation and a willingness to modify treatment. Critically ill SCD patients presenting with presumed infection should undergo thorough echocardiographic examination to determine systolic and diastolic function of both ventricles, the function of cardiac valves, and estimated pulmonary artery pressure.

Circulatory support must be individualized and frequently reassessed. No single inotrope or vasopressor can be recommended, as therapy must be determined on the basis of serial hemodynamic evaluations. Vasoconstrictor medications, though often necessary to maintain vital organ perfusion pressure, should be minimized and weaned whenever possible as they promote sickle hemoglobin polymerization by prolonging transit through the vasculature. Individuals with SCD typically have lower blood pressure than matched African American controls (164), therefore, blood pressure should be viewed in the context of direct and indirect measures of adequate oxygen delivery.

Before permissive hypercapnia and its attendant decrease in pH is adopted, the right ventricle and pulmonary resistance needs to be evaluated, as right heart failure can be precipitated in critically ill patients with SCD. Frequent or continuous assessment of cardiac output, atrial filling pressures, pulmonary artery pressures, and systemic resistance may allow the least harmful ventilatory support to be applied while monitoring for ongoing surveillance of cardiac filling pressures, pulmonary vascular resistance, systemic resistance, and cardiac output. The addition of inhaled nitric oxide may be helpful in supporting right heart function in the face of elevated pulmonary vascular resistance.

Patients with pulmonary artery hypertension are treated with hydroxyurea and chronic transfusion therapy to control the underlying hemolytic state, and oxygen is administered to correct hypoxemia. SCD patients may also be treated with endothelin-1 receptor antagonists (bosentan, ambrisentan) or prostanoids. The use of PDE-5 inhibitors (sildenafil, tadalafil) is not recommended in SCD patients due to frequent hospitalizations for serious adverse events (165).

**Respiratory disease** in the context of fever can be serious and progress rapidly when associated with pneumonia and/or acute chest syndrome. SCD-specific concerns in the setting of pulmonary infection involve the appreciation for the relationship between severe lung disease, elevated pulmonary vascular resistance, and right ventricular dysfunction or failure, as well as the importance of superimposed reactive airway disease as a contributor to deterioration. Analgesia should be provided to allow the patient to cough and breathe deeply if pleuritic chest pain or vaso-occlusive crisis pain is significant. Inhaled nitric oxide, high-frequency oscillatory ventilation, dexamethasone, and extracorporeal membrane oxygenation support have been used to support SCD patients with severe lung disease (166–169). Reactive airway disease should be sought and treated, as this source of ventilation/perfusion mismatch may promote acute chest syndrome. Finally, pulmonary infection can incite acute chest syndrome with increased hospital morbidity and mortality.

**Central nervous system disease** in SCD patients with fever has many serious etiologies. Support of patients with a central nervous system infection is similar to other patients with the added concern for proclivity to stroke. Markedly elevated intracranial pressure is treated with sedation, head of the bed elevation, osmolar therapy with hypertonic saline and mannitol, temperature control, normoglycemia, seizure suppression, and appropriate broad-spectrum antibiotics. Neurosurgical consultation for intracranial pressure monitoring, cerebrospinal fluid drainage, and/or surgical evacuation of blood or purulent material should be considered.

**Aggressive transfusion therapy** can prevent or reverse neurologic injury in patients with SCD (see below). Immediate exchange transfusion to reduce HbS% <30% is protective against stroke, which can be provoked by meningitis, acute chest syndrome, vaso-occlusive crisis, and sepsis. The addition of corticosteroids, which have efficacy in minimizing hearing loss in *H. influenzae* meningitis, but not proven in other forms of meningitis, can be considered as they also have utility in the treatment of acute chest syndrome and reactive airway disease. However, caution is advised, as the use of systemic corticosteroids in SCD is associated with the risk of rebound vaso-occlusive crisis (170).

Aggressive transfusion therapy may be necessary during infection to prevent or treat serious SCD complications, including acute chest syndrome, stroke, acute splenic sequestration, and vaso-occlusive crisis that may occur during or concomitantly with infection and can be life threatening. Simple transfusion (in 10- to 15-mL/kg aliquots) can be administered to optimize oxygen-carrying capacity and minimally changes HbS percentage. Hematocrit should not be increased beyond 30% to avoid an abrupt increase in viscosity that predisposes to stroke. In contrast, exchange transfusion using standard calculations can be performed by automated erythrocytapheresis or manual exchange to quickly decrease the HbS percentage to <30% and increase the hematocrit to approximately 30%. Exchange transfusion can be considered during overwhelming infections to ameliorate and prevent vaso-occlusive crisis, acute chest syndrome, and stroke, and may ameliorate diffuse microvascular occlusion by sickled erythrocytes in patients with multi-organ system failure. Plasma exchange, in addition to red cell exchange, may be helpful for patients with a thrombotic thrombocytopenic purpura-like clinical picture (171).

Although transfusion is regarded as an important therapy to avert serious SCD complications, it is not benign, and repeated transfusions can result in complications such as transfusion-related iron overload, alloimmunization, and delayed hemolytic transfusion reactions, which can result in hyperhemolysis syndrome and death. In many austere environments, the risk of transmission of blood-borne pathogens is prohibitive and precludes transfusion except for the most life-threatening levels of anemia.

The topic of transfusion thresholds frequently arises during the management of persons with SCD who are critically ill. We do not believe that specific thresholds are as helpful as an ongoing evaluation of the risks and benefits of transfusion therapy in the context of the severity of a patient's anemia, adequacy of systemic oxygen delivery, and abundance and safety of the regional blood supply. Simple transfusion of red blood cells should be considered to maintain oxygen-carrying capacity and systemic oxygen delivery and to treat moderate acute chest syndrome. Exchange transfusion should be performed for stroke and severe acute chest syndrome.

**Other hematologic considerations** relate to the support of coagulation abnormalities in severe infection. Coagulopathy is a common finding in serious infections. Clinical judgment is required to determine how aggressively to replete coagulation factors in a disease that is marked by a propensity to both ischemic and hemorrhagic stroke and diffuse vital organ vascular occlusion. In general, they are used sparingly unless coagulation is severely altered or bleeding is present. It is prudent to correct the platelet count, fibrinogen concentration, and coagulation times sufficiently to stop ongoing bleeding, facilitate invasive procedures, and maintain levels above which spontaneous intracranial hemorrhage is likely to occur.

Finally, granulocyte colony-stimulating factor has been used to treat neutropenia and to increase circulating stem cells in other patient populations with few adverse effects. The use of granulocyte colony-stimulating factor in SCD patients is associated with stimulation of severe vaso-occlusive crisis symptoms, acute chest syndrome, multi-organ system failure, and death (172). Further, symptoms have occurred in the SCD population in previously undiagnosed patients and in SCD patients without an elevated neutrophil count. Granulocyte colony-stimulating factor cannot be recommended for use in SCD with sepsis, as catastrophic illness may result. If it is considered for use in a SCD patient, it should only be done when all other therapies have failed and after a complete disclosure of the risk to the patient and family.

## Roadmap for Research and Implementation

Therapies designed to interfere with the sickle cascade at many levels of the pathway are ongoing. Such efforts include strategies to reduce hemoglobin polymerization through the stabilization of HbS with medications such as voxelotor and l-glutamine (173, 174), as well as interference with interactions between the cellular elements of the blood and the endothelium with the monoclonal antibody crizanlizumab (158, 175, 176). Molecular strategies designed to nullify the cellular interactions that characterize the pathophysiology of SCD may someday delay or prevent acquired spleen dysfunction, thus allowing the infant's maturing immune system to develop.

In resource-rich environments, morbidity and mortality related to infectious sequelae have been greatly reduced. In such environments, research should include more effective vaccines to more completely immunize SCD children against bacterial and viral pathogens. Likewise, investigation into barriers that prevent 100% compliance with vaccination and antibiotic prophylaxis is necessary to ensure that children capitalize on the availability of optimal nutrition, disease-modifying medications (hydroxyurea), antibiotic prophylaxis, and vaccination participation. Ongoing surveillance into the development of microbial resistance to antibiotics and immunization strains is needed to stay current with effective prophylaxis and immunization.

In austere environments, policy efforts should continue to emphasize the cost-effective therapies already proven effective elsewhere. These include provision of universal neonatal screening for sickle cell disease, enhanced vaccination, and antibiotic prophylaxis. Delineation of pathogens responsible for viral and bacterial disease in SCD patients in austere environments is necessary, as other pathogens may need to be considered for inclusion for vaccination and antibiotic prophylaxis. Hydroxyurea, an oral disease-modifying medication that is generally effective and well-tolerated, should be made available to as many children with SCD as possible. Finally, efforts in the areas of nutrition, safe blood-banking practices, sanitation, and disease control programs for malaria, tuberculosis, and HIV are essential.

## Strengths and Limitations

The strength of our review is its multidisciplinary approach to the issue of infectious complications that includes the broad perspectives of physicians who specialize in critical care medicine, hematology, and infectious disease.

There are limitations in our review, which include the methodology and the scope of the literature included. This concise review was intended to review pre-determined topics relevant to infectious complications of sickle cell disease. We chose to search the medical literature for case reports, systematic reviews, original research, meta-analyses, narrative reviews, and position/policy statements relevant to SCD and infection. Although we used an extensive combination of terms in our literature search, it is possible that some important references were overlooked. Additionally, we sought only English-language publications, thus limiting our search results.

## Author Contributions

All authors listed have made a substantial, direct and intellectual contribution to the work, and approved it for publication.

### Conflict of Interest

LB has at times received research funding from the National Heart, Lung, and Blood Institute, Pfizer, Micelle BioPharma, HRSA, and Novartis, and has been a consultant for Prolong Pharmaceuticals and Sanofi. The remaining authors declare that the research was conducted in the absence of any commercial or financial relationships that could be construed as a potential conflict of interest.

## Abbreviations

SCD: sickle cell disease
LMIC: low- and middle-income countries
HIC: high-income country.
